# MRI-conditional catheter sensor for contact force and temperature monitoring during cardiac electrophysiological procedures

**DOI:** 10.1186/1532-429X-16-S1-P150

**Published:** 2014-01-16

**Authors:** Yue Chen, Jia Ge, Ka-Wai Kwok, Kent Ronald Nilsson, Mable Fok, Zion T Tse

**Affiliations:** 1 Engineering, University of Georgia, Athens, Georgia, USA; 2 Athens Regional Medical Center, University of Georgia & Georgia Regents University Medical Partnership, Athens, Georgia, USA

## Background

MR-guided cardiac electrophysiological (EP) ablations has drawn increasing attention from both the MRI and EP communities, as high-contrast MR images provide images that couple anatomical information with lesion efficacy [1]. Catheter manipulation can be challenging for cardiac electrophysiologists as conventional electroanatomical maps, frequently include false space. Perforation of heart vessels and chambers by catheters is an uncommon, but devastating, complication during EP procedures arising from either excessive force or vaporization of tissue. Ultimately, these complications arise from an inability to adequately determine catheter-tissue Contact Force (CF) at the catheter tip [2]. Accurate catheter temperature control is of importance during EP Radiofrequency Ablation (RFA) for determining lesion efficacy. We hypothesized that a novel optical sensor design, attachable to a conventional ablation catheter, could allow simultaneous CF and temperature monitoring, providing useful information to the EP physician during the procedure.

## Methods

**Figure 1:**
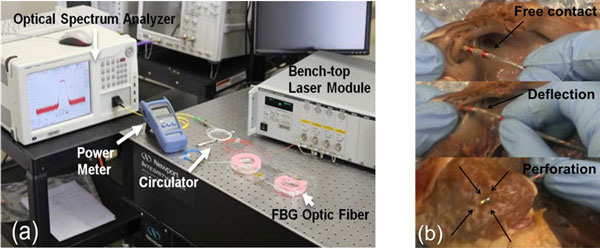
**(a) Experimental setup for characterizing the reflection spectrum and changes in the FBG sensor; (b) Perforation performed in an ex-vivo right atrium with the catheter-tissue CF recorded**.

An optical Fiber Bragg Grating (FBG) sensor was made MR-conditional and installed at the tip of a non-magnetic 8 French EP catheter from St Jude Medical Inc. The FBG sensor was made from an optical fiber, and the sensing signals were transmitted to a measurement setup outside the MRI scanner (Figure 1a). The Wavelength (WL) of light being reflected by the sensor to the measurement equipment depends on the CF and heating that were applied on the catheter tip. The relationship between WL and CF was calibrated with different levels of force that caused catheter deflection and finally perforated an ex-vivo right atrium (Figure 1b). While the relationship between WL and temperature was calibrated on a temperature water bath from 20°C to 80°C with an increment of 10°C per step. Based on the model of compensation between the calibrated CF and temperature [3], both measurements were deduced simultaneously [4].

## Results

**Figure 2:**
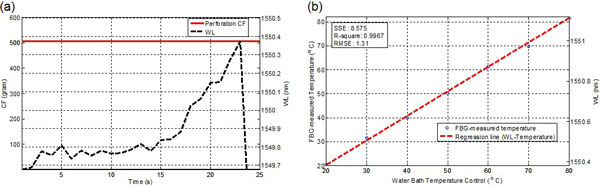
**(a) Measured CF profile during perforation by a catheter instrumented with the FBG sensor**. CF reaches a maximum of 504.5 grams and drops significantly before and after perforation. (b) FBG-measured temperature.

Figure 2a depicts the FBG-measured force profile where the right atrium was perforated by the catheter at 504.5 grams. Figure 2b shows the FBG-measured temperatures aligned with the control temperatures (error < 1.5°C). Both measurements provide useful information for monitoring EP RFA procedure. The catheter FBG sensor unit caused < 5% reduction of Signal to Noise Ratio (SNR) in images taken at 3T MRI, ensuring its compatibility.

## Conclusions

Simultaneous force and temperature monitoring based on the proposed FGB-based sensor design provided useful monitoring in MRI-guided EP therapies.

## Funding

NIH U41-RR019703, R43 HL110427-01, AHA 10SDG261039.
